# Oncological Outcomes after Liver Venous Deprivation for Colorectal Liver Metastases: A Single Center Experience

**DOI:** 10.3390/cancers13020200

**Published:** 2021-01-08

**Authors:** Salah Khayat, Gianluca Cassese, François Quenet, Christophe Cassinotto, Eric Assenat, Francis Navarro, Boris Guiu, Fabrizio Panaro

**Affiliations:** 1 Division of HBP Surgery and Transplantation, Department of Surgery, St. Eloi Hospital, Montpellier University Hospital-School of Medicine, 34090 Montpellier, France; s-khayat@chu-montpellier.fr (S.K.); gianluca.cassese@unina.it (G.C.); f-navarro@chu-montpellier.fr (F.N.); 2 Department of Clinical Medicine and Surgery, University of Naples Federico II, 80131 Napoli, Italy; 3 Department of Surgical Oncology, Cancer Institute of Montpellier (ICM) 208, 34298 Montpellier, France; francois.quenet@icm.unicancer.fr; 4 Division of Interventional Radiology, Department of Radiology, St. Eloi Hospital, Montpellier University Hospital-School of Medicine, 34090 Montpellier, France; c-cassinotto@chu-montpellier.fr (C.C.); b-guiu@chu-montpellier.fr (B.G.); 5 Service d’Oncologie Médicale, CHU Saint Eloi, 34090 Montpellier, France; e.assenat@chu-montpellier.fr

**Keywords:** colorectal liver metastasis, liver venous deprivation (LVD), future liver remnant, oncological outcomes

## Abstract

**Simple Summary:**

In the original retrospective study entitled “Oncological outcomes after Liver Venous Deprivation for Colorectal Liver Metastases: a single center experience” the authors report for the first time the oncological outcomes of Liver Venous Deprivation (LVD) for Colorectal Liver Metastases. LVD is an interventional radiologic technique recently employed before major liver resections and has already showed its safety and effectiveness in inducing contralateral liver hypertrophy. Seventeen consecutive patients undergoing LVD between July 2015 and May 2020 before a right (or extended right) hepatectomy were retrospectively analyzed from an institutional database. The 1-year and 3-year overall survival (OS), as well as hepatic recurrence and Disease Free Survival (DFS), were comparable to literature reports of portal vein embolization (PVE) oncological outcomes.

**Abstract:**

Colorectal liver metastases (CRLM) are the major cause of death in patients with colorectal cancer (CRC). The cornerstone treatment of CRLM is surgical resection. Post-operative morbidity and mortality are mainly linked to an inadequate future liver remnant (FLR). Nowadays preoperative portal vein embolization (PVE) is the most widely performed technique to increase the size of the future liver remnant (FLR) before major hepatectomies. One method recently proposed to increase the FLR is liver venous deprivation (LVD), but its oncological impact is still unknown. The aim of this study is to report first short- and long-term oncological outcomes after LVD in patients undergoing right (or extended right) hepatectomy for CRLM. Seventeen consecutive patients undergoing LVD between July 2015 and May 2020 before an (extended) right hepatectomy were retrospectively analyzed from an institutional database. Post-operative and follow-up data were analyzed and reported. Primary outcomes were 1-year and 3-year overall survival (OS) and hepatic recurrence (HR). Postoperative complications occurred in 8 patients (47%). No deaths occurred after surgery. HR occurred in 9 patients (52.9%). 1-year and 3-year OS were 87% (95% confidence interval [CI]: ±16%) and 60.3%, respectively (95% CI: ±23%). Median Disease-Free Survival (DFS) was 6 months (CI 95%: 4.7–7.2). With all the limitations of a retrospective study with a small sample size, LVD showed similar oncological outcomes compared to literature reports for Portal Vein Embolization (PVE).

## 1. Introduction

Liver resection is considered the mainstay of Colo-Rectal Liver Metastases (CRLM) treatment with excellent long-term oncological outcomes, and low morbidity and mortality [1,2]. In order to minimize post-hepatectomy liver failure (PHLF), a sufficient future liver remnant (FLR) must be preserved [3,4]. Due to inadequate FLR, less than 25% of patients are eligible for surgery [5] at the time of diagnosis [6]. With the intention to optimize the FLR, several techniques have been developed to induce its hypertrophy. Since its introduction in 1984 [7], portal vein embolization (PVE) is nowadays considered the standard technique to induce FLR augmentation [8,9,10]. However, PVE does not always induce sufficient and rapid hypertrophy, yielding a 20% rate of not eligibility to resection due to either insufficient FLR or patient’s dropout for tumor progression [11]. To overcome these limitations, new surgical procedures were recently introduced: the associated liver partition and portal vein ligation for staged hepatectomy (ALPPS) [12], the Radio-frequency-Assisted Liver Partition with Portal vein ligation (RALPP) [13] and the Associating Liver Tourniquet and right Portal occlusion for Staged hepatectomy (ALTPS) [14]. These techniques allow a larger and faster hypertrophy than PVE, but at the cost of significantly greater morbidity and mortality [15]. Over the past 5 years, a new radiological interventional technique of liver venous deprivation (LVD) has emerged. It consists of simultaneous embolization of portal vein and one or two hepatic veins, in order to increase the damage to the liver leading to increased hypertrophy of the contralateral parenchyma [16,17,18], with a kinetic growth rate of 16 ± 7 cc/day according to first reports [19]. Recently, first comparative data have been published regarding FLR volume and function gains [20]. All of them were in favor of greater regeneration after LVD versus PVE. However, the impact of LVD on hepatic recurrence (HR) and long-term oncological outcomes after resection of CLRM remains unknown. There is a considerable interest in this issue, as evidenced by several papers that speculate about negative effects of similar radiological liver augmentation procedures, such as PVE, on tumor growth [21].

The aim of this retrospective study was to report the safety and oncological outcome following major liver resection after LVD in patients with CRLM treated in our center.

## 2. Results

### 2.1. Patients and Tumors Characteristics

Seventeen consecutive patients (*n* = 17) undergoing LVD before right hepatectomy or extended right hepatectomy (trisectionectomy) for Colorectal Liver Metastasis were retrospectively analyzed. In particular, 8 patients received a right hepatectomy, 5 patients received an extended right hepatectomy, 4 received one or more additional wedge resections on the left liver. Patients’ and tumors’ characteristics are shown in Table 1. Mean age was 58.9 (range 39–73). All patients received chemotherapy (CT) before liver surgery. 13 patients (77%) received neoadjuvant CT, 4 (23%) received a conversion surgery strategy. Different CT schemes employed and responses to CT are reported in Table 1. Fourteen patients received post-operative chemotherapy (82.3%) in addition. The median follow-up period was 7.7 months (95% CI: 14.9–45).

### 2.2. Procedures and Liver Volumes

Eight patients received right and median hepatic vein embolization, nine received only right hepatic vein closure. The type of radiological procedure (LVD or extended LVD), FLR volume before and after LVD with the percentage of FLR volume increasing, are shown in Table 2. Median time between LVD and surgery was 39 days (IQR 25–56).

### 2.3. Oncological and Postoperative Outcomes

**Figure 1:**
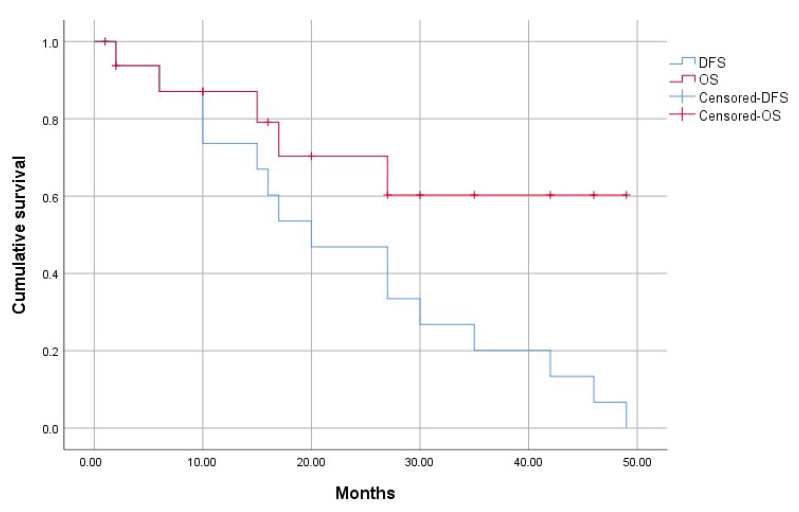
Overall Survival and Disease Free Survival of the patients undergoing LVD for (extended) right hepatectomy for CRLM.3.

**Figure 2:**
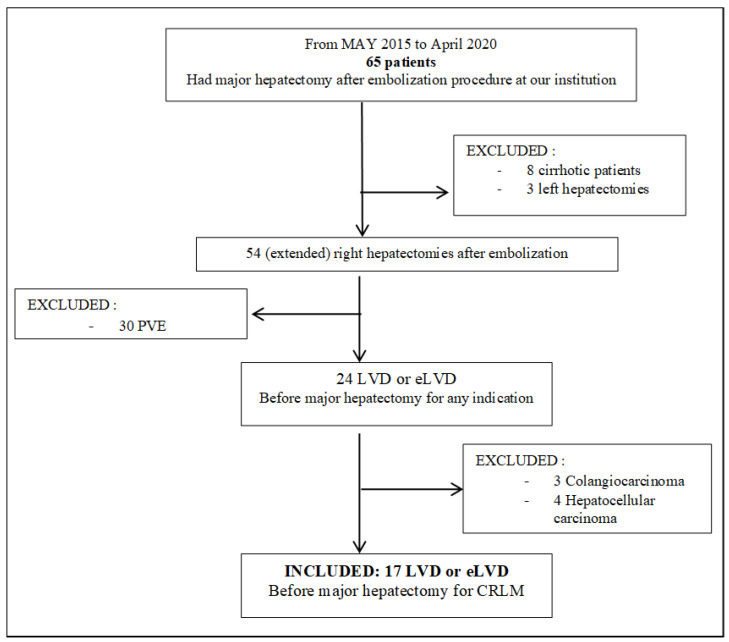
Flow Chart: diagram of patient selection for the retrospective study. LVD: liver venous deprivation; PVE: portal vein embolization; eLVD: extended liver deprivation (portal vein embolization + right and middle hepatic vein embolization); CRLM: colorectal liver metastases.

Hepatic Recurrence occurred in 9 patients (52.9%). No hepatic recurrence underwent further surgical resection. During follow-up, two patients developed a pulmonary progression, two patients a lombo-aortic lymph node metastasis, and one patient a peritoneal carcinosis. Five patients died during follow-up and causes were not related to post-operative or post-procedural events. Overall Survival at 1 year and 3 year were 87% (95% CI: ±16%), and 60.3% (95% CI: ±23%), respectively, (Figure 1). Median DFS was 6 months (CI 95%: 4.7–7.2) (Figure 2). Median time from LVD to surgery was 39 days (IQR_25–75_ 25–56). No patients dropped-out and they all underwent surgery after LVD. No liver progressions after LVD and before resection were observed. Postoperative complications occurred in 8 patients (47%). The severity of complications was recorded as Clavien-Dindo class 1 for two patients, class 2 for five patients and class 3 for one patient that needed an ERCP. In particular, 7 patients reported a post-hepatectomy hemorrhage (41%), all cases of grade one; 1 patient showed a bile leak (5%); 3 patients developed a Post-Hepatectomy Liver Failure of grade A (17%); 4 patients developed post-operative ascites (23.5%), all cases of grade A.

## 3. Materials and Methods

### 3.1. Study Design

This is a single institution observational retrospective study, conducted according to the Strengthening and the Reporting of Observational Studies in Epidemiology (STROBE) guidelines of the EQUATOR network [22]. Informed consent was obtained before both embolization and surgical operation. The study was approved by the institutional review board and registered in clinicaltrial.gov (IRB-MTP_2020_04_202000444, clinicaltrial.gov ID: NCT04370132).

### 3.2. Patients

Seventeen consecutive patients undergoing LVD before right hemi-hepatectomy or extended right hepatectomy between May 2015 and April 2020 were retrospectively analyzed (Figure 2). Cirrhotic patients were excluded. The therapeutic managements of all patients included were previously discussed in a multidisciplinary tumor meeting. The decision to perform a liver augmentation procedure was based on FLR volumetry and functional evaluation based on Tc-99m mebrofenin scintigraphy. The radiological procedure was carried out when expected FLR was <25–30% in normal liver, <35–40% in case of underlying liver disease (cirrhosis, prior chemotherapy and/or cholestasis), or Tc99m mebrofenin extraction below 2.69%/min/m^2^. If both volume and function of the FLR were insufficient, or if liver scintigraphy was unavailable, the radiologists decided to perform the LVD instead of PVE alone, because of the greater volumetric increase shown by the first [23] (Figure 1). Radiological and surgical procedures, as well as patient management, were conducted at the same hospital. Our follow-up policy after LVD was based on contrast-enhanced computed tomography (CT) and Tc99m-Mebrofenin Scintigraphy every week after LVD. The final surgical indication was based on both function and volume data.

### 3.3. Radiological Procedure

The LVD technique has been described in details elsewhere [16]. In summary, right (and accessory right when present) hepatic vein was cannulated under ultrasonographic guidance. The PVE was performed using right transhepatic access. Right portal vessels were embolized using a composite of n-butyl cyanoacrylate and lipiodol (ratio 1:6). The micro-guidewire placed in hepatic vein(s) was then used to roll out an Amplatzer vascular plug II (75% oversizing). The plug is positioned at a distance of about 2 cm from the ostium of the Inferior Vena Cava (IVC), in order to reduce the risk of plug overlength up to IVC. Finally, all distal venous branches were embolized using a mixture of n-butyl cyanoacrylate and lipiodol (ratio 1:6). Eight patients received an embolization of the middle hepatic vein, the so called extended liver venous deprivation (eLVD). The decision whether or not to close the middle hepatic vein was made by the radiologist on the basis of the size of the FLR, of the type of surgery and of the anatomical characteristics of the hepatic veins’ circulation.

### 3.4. Surgical Procedure

Patients included underwent a laparotomic right hemi-hepatectomy (segments 5–8), according to the Brisbane classification of livers resection [24], or extended right hepatectomy, with or without additional wedge resection to the left liver or extension of the hepatectomy to segment 4. An intraoperative ultrasound was performed to confirm the surgical feasibility of the procedure and to guide the resection. The right hepatic artery and portal vein were systematically ligated and transected before the parenchymal transection with an anterior approach. Hepatic veins were closed and divided with a vascular stapled, and the Amplatzer plug was not an obstacle. Pringle maneuver with intermittent clamping and right hepatic vein control were performed if necessary.

### 3.5. Post-Operative Follow-Up

Post-operative and follow-up data were analyzed. Post-operative complications were graded according to Clavien-Dindo classification [25]. PHLF, post-hepatectomy biliary leak (PHBL) and post-hepatectomy hemorrhage (PHH) were diagnosed and classified according to the International Study Group of Liver Surgery (ISGLS) [26,27,28]. Ascites were defined according to the International Ascites Club [29].

All patients were visited in the outpatient department of our center one month after being discharged from the surgery ward. All patients received a clinical, biological and imaging assessment by CT or MRI every 3 months after discharge. The following visits were scheduled every six months if no recurrence was found. In the event of tumor recurrence, the case was re- discussed by the multidisciplinary tumor board with the objective to perform curative treatment as much as possible.

### 3.6. Statistical Analysis

Continuous data are expressed as mean and standard deviation (SD) or median and interquartile range (IQR) depending on whether they have a normal distribution. Categorical data are expressed as frequencies and associated percentages. Primary outcomes were Hepatic Recurrence rate and Overall Survival. Secondary outcomes were disease Free Survival, rate of post-operative complications and the time elapsed between the radiological procedure and surgery. All survival analyses were performed using Kaplan–Meier to compute median and 95% CI. Median follow-up was calculated using reverse Kaplan–Meier methodology. The statistical analysis has been conducted using the SPSS software (version 26.0).

## 4. Discussion

To our knowledge this is the first report on oncological outcomes after LVD for two stage (extended) right hepatectomy for colorectal liver metastases. Previous studies might have suggested negative effects on tumor growth following liver augmentation procedures such as those described in some papers for PVE [21,30,31], therefore may also be after LVD. Such effects may be linked to the increased arterial blood flow, considering that liver metastases have one principal arterial blood supply [32,33]. Furthermore, the LVD procedure has certain technical peculiarities that could have other oncological implications, that deserve to be investigated further.

In our study, oncological results of LVD, which remain preliminary, seem to be comparable to PVE oncological results mainly reported in recent literature. Martinou et al. in a recent single center series reported an OS at 1 year of 68.5%, on 62 patients who underwent PVE. On the other hand, liver metastases from colorectal cancer also confirmed in our study are very prone to recurrence [34]. This recurrence trend is similarly reported in a very recent paper by Bednarsch J. et al., where PVE showed a 3-year OS of 44%, with a median DFS of 10 months, with a sample size of 37 cases. In the same paper, oncological outcomes after ALPPS procedure seem to be likewise comparable to LVD results, with a 3-year OS of 37% and a median DFS of 19 months [35]. Similarly, a very recent meta-analysis comparing ALPPS with a traditional two-stage hepatectomy found a 1-year OS of 79 vs. 84% respectively for ALPPS and PVE [36]. A recent meta-analysis by Giglio et al. has also reported that PVE does not negatively affect oncological outcomes after major liver resections in patients with CRLM [37]. In this paper, no significant differences were found in the incidence of HR, 3-year OS and 5-year OS, when comparing PVE and no PVE groups. We also speculate similar effects and conclusions after LVD procedure, that obviously must be confirmed by further studies.

Another important technique with which our results seem to be comparable is the ultrasound-guided enhanced one-stage hepatectomy (e-OSH) proposed by Torzilli et al. [38]. In patients with CRLM this technique has been shown to combine very low morbidity with excellent oncological outcomes. Data reported on a large cohort of 146 patients and a median follow-up of 39.9 months show an OS of 50% at 3 years, with a 77% global recurrence and a 37.7% hepatic recurrence [39]. At the same time, postoperative outcomes also seem to be comparable, with a reported morbidity of 49.5%, but only 8% for serious complications (very similar to our results, respectively, 47% and 6%). It would be interesting in the future to prospectively compare the two techniques within cohort studies. It is important to underline another aspect reported in our study: the short time elapsed between the LVD procedure and surgery. Various previous papers reported that the delay in hepatic hypertrophy resulting from PVE may itself be one of the main causes of the oncological recurrence reported for the FLR augmentation procedures [40,41]. Similarly, the importance of beginning post-operative chemotherapy as soon as possible following surgery is well known, and this time is indirectly related to FLR regeneration time, as well as to the postoperative course. Our data showed a median time between LVD and surgery of 39 days (IQR_25–75_ 25–56), that is comparable to PVE reports from literature [8]. This delay might be regarded as too long for a procedure supposed to hypertrophy the FLR faster. We must acknowledge that this delay could be significantly shortened since both volume and function hypertrophy of the FLR have been shown to occur as soon as 7 days after LVD [20]. Efforts should be made to shorten surgery planning, which remains difficult in daily practice especially in the context of COVID-19 pandemic with less resources.

Furthermore, the LVD technique has been shown in our series to be feasible, well tolerated and to provide an important augmentation of the FLR volume for right hepatectomy, with acceptable morbidity and mortality. Previous data on perioperative surgical outcomes had already been reported by our group [19]. In the previous paper data from LVD and PVE before major hepatectomies were compared, but in a smaller sample of patients with any indication. Our current study, on the other hand, analyzes this time long-term oncological and surgical safety data on a sample of patients homogeneous by pathology.

Finally, it is particularly important that all the patients who underwent LVD for CRLM at our institution also underwent surgery, with no patients lost due to post-procedural complications or tumor progression. These are very encouraging data when compared to PVE, even if only from a preliminary report with a very small sample, and which can be affected by various confounding factors. It should be noted that similar results on the dropout rate of patients with LVD were also reported by Kobayashi et coll. Ref. [42], in a comparative study with PVE on a sample composed mostly of patients with CRLM [43].

This study has several limitations. First, it is observational and retrospective in nature, with a purely descriptive statistic. Due to the recent nature of this technique, few consecutive patients were included in this study and they presented with a heterogenous subset of liver and oncological characteristics. The choice to publish this type of report arose from the scarcity of LVD cases, that would have required another type of design and work in order to have a significance in statistical inference, together with the interest that this type of positive results aroused in the center that first proposed and carried out this type of technique. The small sample size of this series does not allow a significant comparison with a control group or a detailed analysis on which factors can play a prognostic role on both OS and DFS. Furthermore, all the results, both oncological and not, are very encouraging and deserve to be shared to arouse interest and enthusiasm on a topic that is still much debated. It will be important to update the oncological data over the next few years to have results after 5 years of follow-up, possibly including new patients. Randomized controlled trials (RCT) are needed to confirm the benefit of LVD. Actually, one RCT (promoted by our team) started in France (HYPER-LIV 01), and another International RCT called “DRAGON-1” is a work in progress.

## 5. Conclusions

In conclusion, this observational study, despite the small number, reports very encouraging oncological results regarding the LVD technique, also in consideration of the recent introduction of the procedure that may be improved. Data regarding procedure tolerability, postoperative morbidity and mortality, as well as the time between procedure and surgery and the percentage of patients undergoing surgery for CRLM after LVD, are also promising, and we are waiting for large-scale randomized data.

## Figures and Tables

**Table 1:** Patients’ and tumors’ characteristics. RECIST: Response Evaluation Criteria in Solid Tumours; CT: Chemotherapy; FOLFOX: Folinic acid, 5-Fluorouracil, Oxaliplatin; FOLFIRI: Folinic acid, 5-Fluorouracil, Irinotecan. FOLFIRINOX: Folinic acid, 5-Fluorouracil, Oxaliplatin, Irinotecan; EGFR: Epidermal growth factor receptor.

Age (Mean, Range)	58.9 (39–73)
Sex (M/F)	13/4
Primary tumor localization	
Right colon	5 (29.5%)
Left colon	11(64.5%)
Rectum	1 (6%)
Liver metastasis presentation	
synchronous	14 (82.5%)
Metachronous	3 (17.5%)
First CRC stadium	
T1N1M0	1 (6%)
T3N0M0	1 (6%)
T3N1M1	4 (24%)
T3N2M1	7 (46%)
T4N1M0	2 (12%)
T4N2M1	2 (12%)
Chemotherapy before surgery	17 (100%)
Response to preoperative chemotherapy according to RECIST criteria	
Partial Response	5 (30%)
Stable Disease	12 (70%)
Chemotherapy cycles before liver surgery (Median (IQRs_25–75_)	6 (4–14)
First line chemotherapy schemes	17
FOLFOX	1 (6%)
FOLFIRI	3 (17.5%)
FOLFIRI + CETUXIMAB	3 (17.5%)
FOLFIRINOX	4 (24%)
FOLFOXIRI + BEVACIZUMAB	3 (17.5%)
FOLFOX + BEVACIZUMAB	2 (12%)
FOLFIRI + BEVACIZUMAB	1 (6%)
Second line chemotherapies	6
FOLFOXIRI + BEVACIZUMAB	1 (16.5%)
FOLFOXIRI + CETUXIMAB	2 (34%)
FOLFIRI + BEVACIZUMAB	1 (16.5%)
FOLFOX	1 (16.5%)
5FU + BEVACIZUMAB	1 (16.5%)
Third line chemotherapy	1
FOLFIRI + BEVACIZUMAB	1 (100%)
Liver First surgical strategy (*n*)	4 (23.5%)
Post-chemotherapy fibrosis or steatosis > 60%	2 (12%)
Prior liver resection	9 (52%)
Prior percutaneous thermal ablation	5 (29.5%)
Time between CT and Surgery (Median, IQR_25–75_)	64 (59.5–90)
Time between LVD and Surgery (Median, IQR_25–75_)	39 (25–56)
Extended right hepatectomies	8 (47%)

**Table 2:** Liver volumes before and after liver venous deprivation (LVD). Median time between LVD and surgery was 39 days (IQR_25–75_ 25–56).

Liver Volumes	Before LVD	After LVD (7–10 Days Before Surgery)
Total Liver Volume (Median, IQR)	1803 mL (1496.5–2031)	2025 mL (1782–2191.5)
Future Liver Remnant Volume	451 mL (408.5–602)	761 mL (566–914)
% of FLR	29% (23.5–33)	39% (35.5–45.3)

## Data Availability

The data presented in this study are available on request from the corresponding author. The data are not publicly available due to privacy reasons.

## References

[1] Choti M.A., Sitzmann J.V., Tiburi M.F., Sumetchotimetha W., Rangsin R., Schulick R.D., Lillemoe K.D., Yeo C.J., Cameron J.L. (2002). Trends in long-term survival following liver resection for hepatic colorectal metastases. Ann. Surg..

[2] Rees M., Tekkis P.P., Welsh F.K.S., O’Rourke T., John T.G. (2008). Evaluation of Long-term Survival After Hepatic Resection for Metastatic Colorectal Cancer. Ann. Surg..

[3] Xu F., Tang B., Jin T.Q., Dai C.L. (2018). Current status of surgical treatment of colorectal liver metastases. World J. Clin. Cases.

[4] Schindl M.J., Redhead D.N., Fearon K.C.H., Garden O.J., Wigmore S.J. (2005). The value of residual liver volume as a predictor of hepatic dysfunction and infection after major liver resection. Gut.

[5] Khatri V.P., Petrelli N.J., Belghiti J. (2005). Extending the frontiers of surgical therapy for hepatic colorectal metastases: Is there a limit?. J. Clin. Oncol..

[6] Kubota K., Makuuchi M., Kusaka K., Kobayashi T., Miki K., Hasegawa K., Harihara Y., Takayama T. (1997). Measurement of liver volume and hepatic functional reserve as a guide to decision-making in resectional surgery for hepatic tumors. Hepatology.

[7] Makuuchi M., Thai B.L., Takayasu K., Takayama T., Kosuge T., Gunvén P., Yamazaki S., Hasegawa H., Ozaki H. (1990). Preoperative portal embolization to increase safety of major hepatectomy for hilar bile duct carcinoma: A preliminary report. Surgery.

[8] Van Lienden K.P., Van Den Esschert J.W., De Graaf W., Bipat S., Lameris J.S., Van Gulik T.M., Van Delden O.M. (2013). Portal vein embolization before liver resection: A systematic review. Cardiovasc. Intervent. Radiol..

[9] Glantzounis G.K., Tokidis E., Basourakos S.P., Ntzani E.E., Lianos G.D., Pentheroudakis G. (2017). The role of portal vein embolization in the surgical management of primary hepatobiliary cancers. A systematic review. Eur. J. Surg. Oncol..

[10] Abulkhir A., Limongelli P., Healey A.J., Damrah O., Tait P., Jackson J., Habib N., Jiao L.R. (2008). Preoperative portal vein embolization for major liver resection: A meta-analysis. Ann. Surg..

[11] Alvarez F.A., Castaing D., Figueroa R., Allard M.A., Golse N., Pittau G., Ciacio O., Sa Cunha A., Cherqui D., Azoulay D. (2018). Natural history of portal vein embolization before liver resection: A 23-year analysis of intention-to-treat results. Surgery (United States).

[12] Alvarez F.A., Ardiles V., de Santibañes M., Pekolj J., de Santibañes E. (2014). Associating Liver Partition and Portal Vein Ligation for Staged Hepatectomy Offers High Oncological Feasibility with Adequate Patient Safety: A Prospective Study at a Single Center. Ann. Surg..

[13] Gall T.M.H., Sodergren M.H., Frampton A.E., Fan R., Spalding D.R., Habib N.A., Pai M., Jackson J.E., Tait P., Jiao L.R. (2015). Radio-frequency-assisted Liver Partition with Portal Vein Ligation (RALPP) for Liver Regeneration. Ann. Surg..

[14] Robles R., Parrilla P., López-Conesa A., Brusadin R., de la Peña J., Fuster M., García-López J.A., Hernández E. (2014). Tourniquet modification of the associating liver partition and portal ligation for staged hepatectomy procedure. Br. J. Surg..

[15] Kang D., Schadde E. (2017). Hypertrophy and Liver Function in ALPPS: Correlation with Morbidity and Mortality. Visc. Med..

[16] Guiu B., Chevallier P., Denys A., Delhom E., Pierredon-Foulongne M.A., Rouanet P., Fabre J.M., Quenet F., Herrero A., Panaro F. (2016). Simultaneous trans-hepatic portal and hepatic vein embolization before major hepatectomy: The liver venous deprivation technique. Eur. Radiol..

[17] Hwang S., Ha T.Y., Ko G.Y., Kwon D.I., Song G.W., Jung D.H., Kim M.H., Lee S.K., Lee S.G. (2015). Preoperative Sequential Portal and Hepatic Vein Embolization in Patients with Hepatobiliary Malignancy. World J. Surg..

[18] Madoff D.C., Odisio B.C., Schadde E., Gaba R.C., Bennink R.J., van Gulik T.M., Guiu B. (2020). Improving the Safety of Major Resection for Hepatobiliary Malignancy: Portal Vein Embolization and Recent Innovations in Liver Regeneration Strategies. Curr. Oncol. Rep..

[19] Panaro F., Giannone F., Riviere B., Sgarbura O., Cusumano C., Deshayes E., Navarro F., Guiu B., Quenet F. (2019). Perioperative impact of liver venous deprivation compared with portal venous embolization in patients undergoing right hepatectomy: Preliminary results from the pioneer center. HepatoBiliary Surg. Nutr..

[20] Guiu B., Quenet F., Panaro F., Piron L., Cassinotto C., Herrerro A., Souche F.-R., Hermida M., Pierredon-Foulongne M.-A., Belgour A. (2020). Liver venous deprivation versus portal vein embolization before major hepatectomy: Future liver remnant volumetric and functional changes. Hepatobiliary Surg. Nutr..

[21] Pamecha V., Levene A., Grillo F., Woodward N., Dhillon A., Davidson B.R. (2009). Effect of portal vein embolisation on the growth rate of colorectal liver metastases. Br. J. Cancer.

[22] Cuschieri S. (2019). The STROBE guidelines. Saudi J. Anaesth..

[23] Guiu B., Quenet F., Escal L., Bibeau F., Piron L., Rouanet P., Fabre J.M., Jacquet E., Denys A., Kotzki P.O. (2017). Extended liver venous deprivation before major hepatectomy induces marked and very rapid increase in future liver remnant function. Eur. Radiol..

[24] Strasberg S.M., Phillips C. (2013). Use and dissemination of the Brisbane 2000 nomenclature of liver anatomy and resections. Ann. Surg..

[25] Dindo D., Demartines N., Clavien P.-A. (2004). Classification of Surgical Complications. Ann. Surg..

[26] Rahbari N.N., Garden O.J., Padbury R., Brooke-Smith M., Crawford M., Adam R., Koch M., Makuuchi M., Dematteo R.P., Christophi C. (2011). Posthepatectomy liver failure: A definition and grading by the International Study Group of Liver Surgery (ISGLS). Surgery.

[27] Rahbari N.N., Garden O.J., Padbury R., Maddern G., Koch M., Hugh T.J., Fan S.T., Nimura Y., Figueras J., Vauthey J.N. (2011). Post-hepatectomy haemorrhage: A definition and grading by the International Study Group of Liver Surgery (ISGLS). HPB.

[28] Koch M., Garden O.J., Padbury R., Rahbari N.N., Adam R., Capussotti L., Fan S.T., Yokoyama Y., Crawford M., Makuuchi M. (2011). Bile leakage after hepatobiliary and pancreatic surgery: A definition and grading of severity by the International Study Group of Liver Surgery. Surgery.

[29] Moore K.P., Wong F., Gines P., Bernardi M., Ochs A., Salerno F., Angeli P., Porayko M., Moreau R., Garcia-Tsao G. (2003). The management of ascites in cirrhosis: Report on the consensus conference of The International Ascites Club. Hepatology.

[30] Kokudo N., Tada K., Seki M., Ohta H., Azekura K., Ueno M., Ohta K., Yamaguchi T., Matsubara T., Takahashi T. (2001). Proliferative activity of intrahepatic colorectal metastases after preoperative hemihepatic portal vein embolization. Hepatology.

[31] Hoekstra L.T., van Lienden K.P., Doets A., Busch O.R.C., Gouma D.J., van Gulik T.M. (2012). Tumor progression after preoperative portal vein embolization. Ann. Surg..

[32] Nagino M., Nimura Y., Kamiya J., Kanai M., Hayakawa N., Yamamoto H. (1998). Immediate increase in arterial blood flow in embolized hepatic segments after portal vein embolization: CT demonstration. AJR Am. J. Roentgenol..

[33] Archer S.G., Gray B.N. (1989). Vascularization of small liver metastases. Br. J. Surg..

[34] Martinou E., Kostalas M., Kumar R., Riga A., Worthington T., Horton A., Karanjia N. (2018). Oncological outcomes of portal vein embolisation followed by major liver resection: A 6-year experience. HPB.

[35] Bednarsch J., Czigany Z., Sharmeen S., Van Der Kroft G., Strnad P., Ulmer T.F., Isfort P., Bruners P., Lurje G., Lurje G. (2020). ALPPS versus two-stage hepatectomy for colorectal liver metastases—A comparative retrospective cohort study. World J. Surg. Oncol..

[36] Zhang L., Yang Z., Zhang S., Wang W., Zheng S. (2020). Conventional Two-Stage Hepatectomy or Associating Liver Partitioning and Portal Vein Ligation for Staged Hepatectomy for Colorectal Liver Metastases? A Systematic Review and Meta-Analysis. Front. Oncol..

[37] Giglio M.C., Giakoustidis A., Draz A., Jawad Z.A.R., Pai M., Habib N.A., Tait P., Frampton A.E., Jiao L.R. (2016). Oncological Outcomes of Major Liver Resection Following Portal Vein Embolization: A Systematic Review and Meta-analysis. Ann. Surg. Oncol..

[38] Torzilli G., Adam R., Vigano L., Imai K., Goransky J., Fontana A., Toso C., Majno P., De Santibanesc E. (2017). Surgery of colorectal liver metastases: Pushing the limits. Liver Cancer.

[39] Torzilli G., Viganò L., Gatti A., Costa G., Cimino M., Procopio F., Donadon M., Del Fabbro D. (2017). Twelve-year experience of “radical but conservative” liver surgery for colorectal metastases: Impact on surgical practice and oncologic efficacy. HPB.

[40] Simoneau E., Aljiffry M., Salman A., Abualhassan N., Cabrera T., Valenti D., El Baage A., Jamal M., Kavan P., Al-Abbad S. (2012). Portal vein embolization stimulates tumour growth in patients with colorectal cancer liver metastases. HPB.

[41] Beal I.K., Anthony S., Papadopoulou A., Hutchins R., Fusai G., Begent R., Davies N., Tibballs J., Davidson B. (2006). Portal vein embolisation prior to hepatic resection for colorectal liver metastases and the effects of periprocedure chemotherapy. Br. J. Radiol..

[42] Kobayashi K., Yamaguchi T., Denys A., Perron L., Halkic N., Demartines N., Melloul E. (2020). Liver venous deprivation compared to portal vein embolization to induce hypertrophy of the future liver remnant before major hepatectomy: A single center experience. Surgery (United States).

[43] Luz J.H.M., Bilhim T., Gomes F.V., Costa N.V., Coimbra E. (2020). Regarding: “Liver venous deprivation compared with portal vein embolization to induce hypertrophy of the future liver remnant before major hepatectomy: A single center experience”. Surgery (United States).

